# The regulation and function of inositol 1,4,5-trisphosphate 3-kinases

**DOI:** 10.1016/j.advenzreg.2006.01.009

**Published:** 2006

**Authors:** Robin F. Irvine, Samantha M. Lloyd-Burton, Jowie C.H. Yu, Andrew J. Letcher, Michael J. Schell

**Affiliations:** Department of Pharmacology, Tennis Court Road, Cambridge CB2 1PD, UK

## Introduction

The specific phosphorylation of inositol 1,4,5-trisphosphate (Ins(1,4,5)*P*_3_) in the 3-position (EC 2.7.1.127) was discovered 20 yr ago (Batty et al., 1985; Irvine et al., 1986a, b). At that time it represented an unexpected complication in the metabolism of Ins(1,4,5)*P*_3_, whose second messenger function was just emerging (Streb et al., 1983; Berridge and Irvine, 1984). Moreover, given that we already knew that Ins(1,4,5)*P*_3_ can be inactivated by a specific 5-phosphatase (EC 3.1.3.56) (Downes et al., 1982), it was something of a puzzle as to why cells should also consume ATP to phosphorylate Ins(1,4,5)*P*_3_. This conundrum was further enhanced when we found that Ins(1,3,4,5)*P*_4_ did not mobilize Ca^2+^ (Irvine et al., 1986a, b) so, superficially, Ins(1,4,5)*P*_3_ 3-kinase (IP_3_3 K) could indeed be viewed as just a ‘redundant’ off-switch for Ins(1,4,5)*P*_3_.

In the ensuing time, we have learned an enormous amount about the three members of the mammalian IP_3_3K family (for reviews of their overall molecular biology and properties see Communi et al. (1995); Irvine and Schell (2001); Pattni and Banting (2004)). We have also gone a long way towards an understanding of the ‘why’ question—why do cells bother to make Ins(1,3,4,5)*P*_4_, and consume an ATP molecule, when they have a perfectly good 5-phosphatase to inactivate Ins(1,4,5)*P*_3_? The answer to this question is a long and complicated one, and its principal components lie firstly in our knowledge of the evolution of the IP_3_3Ks and, secondly, in what we know about their localization and regulation.

### Evolution of Ins(1,4,5)*P*_3_ 3-kinases

One of our major sources of enlightenment has stemmed from an understanding of where IP_3_3Ks came from. We now know that IP_3_3K is a member of a larger family of enzymes that began early in eukaryotic evolution (Irvine and Schell, 2001; Irvine, 2005), either as ‘Ins*P*_3_ multi-kinases’ (EC 2.7.1.151, i.e., inositol phosphate kinases that can phosphorylate several substrates), or Ins*P*_6_ kinases (EC 2.7.4.21). Together these enzymes form a major part of a route of synthesis of the pyro-phosphorylated inositol phosphates Ins(*PP*)*P*_5_ and Ins(*PP*)_2_*P*_4_ (more loosely known as Ins*P*_7_ and Ins*P*_8_, respectively). Discussion of these pathways and their functions can be found in, e.g., Irvine and Schell (2001); Shears (2001); Irvine (2005); York et al. (2005), and we return to them at the end of this review. Note that a particularly intriguing possible function for Ins(*PP*)*P*_5_ has emerged recently with the suggestion that it may directly (and non-enzymatically) phosphorylate some proteins, particularly proteins found in the nucleolus (Saiardi et al., 2004).

**Fig. 1:**
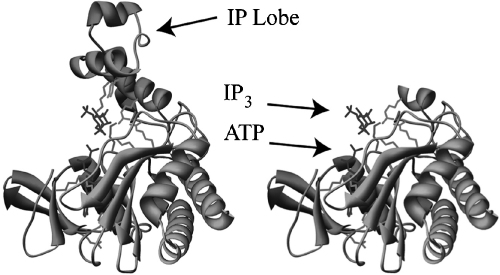
Ins(1,4,5)*P*_3_ binding to IP_3_KA. This illustration is derived from the structure of IP_3_KA with Ins(1,4,5)*P*_3_ and ATP bound to it, as described by Gonzalez et al. (2004). On the left is the original structure, and on the right is exactly the same, but with the three-helices insert (IP lobe) that is unique to the Ins(1,4,5)*P*_3_ 3-kinases (Gonzalez et al., 2004) removed. Thus, the structure on the right envisions how Ins(1,4,5)*P*_3_ might bind to an Ins*P*_3_ multikinase (lacking these three helices) which could, therefore, enable a wider range of substrates binding, including PtdIns(4,5)*P*_2_ (Resnick et al., 2005).

From this recent cloning activity we can see that the IP_3_3Ks evolved out of an inositol phosphate kinase family late in eukaryotic evolution, probably about the time when metazoans emerged (Irvine and Schell, 2001). Moreover, from a comparison of the primary sequences of the family viewed in the context of how IP_3_3KA binds its Ins(1,4,5)*P*_3_ substrate with such exquisite specificity (Gonzalez et al., 2004), we can see that the crucial evolutionary change was the insertion of three extra *α*-helices in the Ins*P*-binding domain. This insertion holds the Ins(1,4,5)*P*_3_-binding residues in a precise orientation (see Fig. 1), and ensures an absolute specificity for the substrate, including not least ensuring that it will not phosphorylate PtdIns(4,5)*P*_2_ to generate the (already established when IP_3_3Ks evolved) second messenger PtdIns(3,4,5)*P*_3_ (Gonzalez et al., 2004; Miller and Hurley, 2004); note the contrast (Fig. 1) with the related ‘Ins*P*_3_ multikinase’, which lacks these three *α*-helices (Gonzalez et al., 2004), and can phosphorylate PtdIns(4,5)*P*_2_ to PtdIns(3,4,5)*P*_3_ (Resnick et al., 2005). So, as the first IP_3_3K emerged in the form of a specific Ins(1,4,5)*P*_3_ 3-kinase, why did evolution seize on it, keep it and use it extensively? The answer to this lies in the three possible functions of IP_3_3Ks, and it is these functions, and the regulation of the IP_3_3Ks’ location and activity, that are the subject of the rest of this paper.

In simple terms, there are three possible functions for IP_3_3Ks: (a) to remove Ins(1,4,5)*P*_3_, thus terminating its second messenger function; (b) to synthesize Ins(1,3,4,5)*P*_4_, which acts as a second messenger in its own right; and (c) to serve as the first step in a route of synthesis of the higher inositol phosphates.

### Functions (a)—removal of Ins(1,4,5)*P*_3_

As hinted at above, to avoid redundancy of IP_3_3K with Ins(1,4,5)*P*_3_ 5-phosphatase as a remover of Ins(1,4,5)*P*_3_, we would expect there to be clear differences between the two enzymes in terms of their physiology. Indirect evidence for this comes from the different phenotypes engendered in *Caenorhabditis elegans* when the IP_3_3K (Clandinin et al., 1998) versus the Ins(1,4,5)*P*_3_ 5-phosphatase (Bui and Sternberg, 2002) are knocked out. The first difference in actual properties between the two enzymes to be discovered was that IP_3_3K has a higher affinity but lower *V*_max_ than Ins(1,4,5)*P*_3_ 5-phosphatase (Irvine et al., 1986a, b; Connolly et al., 1987), implying that perhaps IP_3_3K is the principal route of Ins(1,4,5)*P*_3_ metabolism in cells, with the phosphatase being there to remove rapidly any excess Ins(1,4,5)*P*_3_ (a relationship reminiscent of that between the high-affinity, low-capacity Ca^2+^-ATPases, and the low-affinity, high-capacity Ca^2+^/Na^+^ exchangers). One might, therefore, expect that at low doses of agonists (and thus at low Ins(1,4,5)*P*_3_ levels) the route of Ins(1,4,5)*P*_3_ metabolism is mostly by the kinase, and some data on inositol phosphates generated support that suggestion indirectly. But interestingly, in mice with either IP_3_3KA (Jun et al., 1998) or IP_3_3KB (Pouillon et al., 2003) knocked out, the authors reported no difference in Ins(1,4,5)*P*_3_ levels in relevant tissues, whereas in cells with reduced Ins(1,4,5)*P*_3_ 5-phosphatase, the authors documented increased Ins(1,4,5)*P*_3_ levels, leading to a change in cellular physiology (Speed et al., 1999). These issues may be complicated by the possible existence of multiple pools of Ins(1,4,5)*P*_3_ (see (Irvine and Schell, 2001 for discussion) and, overall, we still have no clear answer to the relative contributions of the two enzymes to Ins(1,4,5)*P*_3_ removal; it may be that it varies between tissues and organisms. More interesting and compelling evidence for a separation of the functions of the two enzymes lies in their very different regulation and localization.

At an early stage in its evolution, some time between the emergence of nematodes and arthropods (Irvine and Schell, 2001), IP_3_3K became regulated by Ca^2+^/calmodulin (Communi et al., 1995). Later (in vertebrates), some IP_3_3K isoforms became activatable by phosphorylation on a threonine residue that is close to the catalytic site (Gonzalez et al., 2004) by CaMKII (Communi et al., 1997, 1999). This is in marked contrast to the inhibition of Ins(1,4,5)*P*_3_ 5-phosphatase caused by CaMKII phosphorylation (Communi et al., 2001). Thus, an activation of CaMKII will divert Ins(1,4,5)*P*_3_ specifically towards Ins(1,3,4,5)*P*_4_, which might be taken as indirect evidence for some specific function of the latter (see below).

Another factor that separates the kinase and phosphatase is their distinctly different localization. The Ins(1,4,5)*P*_3_ 5-phosphatase is localized to the plasma membrane by being farnesylated (De Smedt et al., 1997). However, the A and B isoforms of IP_3_3K are targeted either to the actin skeleton or to the endoplasmic reticulum (e.r.), and this targeting and its regulation represents a crucial aspect of their physiological function.

### Localization of IP_3_3Ks

The localization of the C isoform of IP_3_3K is controversial in that one group have claimed a partially nuclear (and dynamic) localization (Nalaskowski et al., 2003), and the other a cytosolic localization (Dewaste et al., 2003). We will not discuss this issue further, but will instead focus on the localization of the A and B isoforms, which is more fully understood.

IP_3_3KA is confined to the testis and to neurons, and in neurons its localization is dynamic and under tight regulation. IP_3_3KA is expressed at highest levels in the cerebellum and the hippocampus, especially in the neuropil of the CA1 and dentate gyrus regions of the latter (Mailleux et al., 1991; Schell and Irvine, 2006). Moreover, this high level of enzyme is further concentrated by a clear localization to the post-dendritic spines of the neurons, a localization that is effected by a specific and novel F-actin binding domain consisting of the N-terminal 66 amino acids of IP_3_3KA (Schell et al., 2001). In such a position, where it lies between the major site of Ins(1,4,5)*P*_3_ generation (by the post-synaptic density) and the site of its action (the e.r.), IP_3_3KA is perfectly poised to act as a sort of ‘firewall’ (Schell et al., 2001), dictating by its activity the degree to which Ins(1,4,5)*P*_3_ can ‘escape’ from the spine and mobilize Ca^2+^ (Schell et al., 2001). The acute and long-term regulation of IP_3_3KA by Ca^2+^ that is discussed above can then be viewed as a way in which stimulation of a spine to increase its Ca^2+^ will in turn dictate how it regulates the actions of Ins(1,4,5)*P*_3_.

We have explored this activation by Ca^2+^ in transfected cells (SML-B, JCHY, RFI and MJS, unpublished), and shown that even in a simple system like a HeLa cell, the influence of calmodulin and CaMKII is detectable in the ability of IP_3_3KA to decrease Ins(1,4,5)*P*_3_-induced Ca^2+^ mobilization. Moreover, the localization to the actin skeleton also has a clear effect on the efficacy of IP_3_3KA, again, even in such an unpolarized cell as a HeLa (SML-B, JCHY, RFI and MJS, unpublished), so when we consider such a highly polarized structure as a dendrite, the localization of IP_3_3KA adds a new dimension to the inter-relationship between the various stimulatory inputs into postsynaptic spines (Schell et al., 2001).

**Fig. 2:**
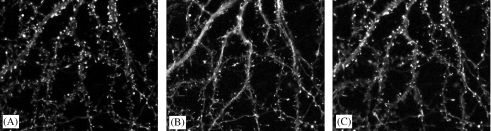
Dynamic localization of Ins(1,4,5)*P*_3_ 3-kinase A in postsynaptic spines. The figure shows IP_3_KA transfected into hippocampal neurones, and illustrates (A) before, (B) 2 min after, treatment with 100 μM glutamate, and (C) 20 min after removal of 100 μM glutamate and the addition of MK-801, an NMDA receptor antagonist. The reversible shift from the spines to the shaft induced by glutamate is plainly visible. See (Schell and Irvine, 2006) for all details.

Our more recent investigations of IP_3_3KA have now added another crucial dimension to this complexity, the dimension of time. Firstly, by using IP_3_3KA as a sort of real-time marker for F-actin, we have revealed that the F-actin in spines is in a highly dynamic state. As far as we can tell it is not turning over very rapidly, but rather the entire F-actin is in a state of dynamic equilibrium, whereby Ca^2+^ entry (especially through NMDA receptors) is driving it as a single entity out of the spines and into the main dendritic shaft (Fig. 2 and (Schell and Irvine, 2006)), carrying IP_3_3KA with it. This is a physiological response in so far as resting synaptic activity has a significant effect on IP_3_3KA localization (Schell and Irvine, 2006). Moreover, at high levels of stimulation with exogenous glutamate, which could be taken as a mimicking of massive stimulation such as follows ischemic shock, the exit of IP_3_3KA is total and rapid (within seconds), and may form the first stage in the pronounced changes in spine structure that follow such events (Halpain et al., 1998). It is important to note, however, that provided the stimulation with glutamate is not too prolonged, it remains fully reversible within about 20 min (Fig. 2 and (Schell and Irvine, 2006)). It remains to be seen what the functional consequences of this movement of IP_3_3KA are—as we have discussed elsewhere (Schell and Irvine, 2006); both removing IP_3_3KA from the spines and placing it in the shafts will have profound consequences for Ins(1,4,5)*P*_3_-mediated Ca^2+^ signalling. Moreover, as the principal Ins(1,4,5)*P*_3_-generating receptors in this system (metabotropic and muscarinic) have a minimal influence on IP_3_3KA movement, another consequence of these discoveries is to expand the complexity of the relationship between the different types of glutamate receptors.

Secondly, an extra layer of complexity in the dimension of time can be found in the spatial relationship between the e.r. and the actin skeleton. Our three-dimensional reconstruction of images (Schell and Irvine, 2006) has confirmed the close spatial relationship between these structures in those spines into which the e.r. penetrates (Capani et al., 2001). However, all these images depict motionless structures, and the picture changes when we think about how spines themselves are moving (Fischer et al., 1998), and that movement itself impinges on the Ca^2+^ kinetics within the spine (Majewska et al., 2000). Moreover, we have shown that the e.r. itself is a highly motile organelle whose movements are under acute regulation by Ca^2+^ in both ATP-dependent and independent mechanisms (Brough et al., 2005a, b). Taking this remarkable complexity concerning IP_3_3KA and the actions of Ins(1,4,5)*P*_3_ in dendrites overall, we can begin to see that there is no functional redundancy with Ins(1,4,5)*P*_3_ 5-phosphatase—in parallel experiments to those described above (Schell and Irvine, 2006), Ins(1,4,5)*P*_3_ 5-phosphatase does not localize to dendritic spines, but is instead distributed all along the plasma membrane of the dendrite, and it does not move perceptibly when glutamate is added (MJS and RFI, unpublished). Thus, it makes every sense that a primary function of IP_3_3KA could be to remove Ins(1,4,5)*P*_3_.

Finally, a brief consideration of IP_3_3KB supports this conclusion. Our understanding of the regulation of this isoform is not quite so advanced as for IP_3_3KA but, nevertheless, we know that in addition to its dual stimulation by CaM and CaMKII (Communi et al., 1999), IP_3_3KB also has an interesting localization as a part of its function. Originally, it was described as being either cytosolic or in the e.r. (Soriano et al., 1997), but subsequent cloning of the full length enzyme (the original cloning was missing some of the N-terminus) has led to the realization that it is partially localized to the cytoskeleton (Dewaste et al., 2003), because it too has an actin-binding domain (Brehm et al., 2004). The localization in the e.r. is due to a distinct part of the N-terminus (Pattni et al., 2003), and we have recently shown that removal of the first 150 amino acids from the N-terminus of IP_3_3KB causes the enzyme to move from the actin skeleton to the e.r. (Yu et al., 2005). Moreover, this localization is subject to regulation by proteolytic cleavage (Pattni et al., 2003; Yu et al., 2005), an event that we have shown takes place under physiological conditions, at least with the transfected enzyme (Yu et al., 2005). Finally, as with the A-isoform (SML-B, JCHY, RFI and MJS, unpublished), the localization of the enzyme alters its ability to metabolize Ins(1,4,5)*P*_3_, as assayed by decreases in Ins(1,4,5)*P*_3_-induced Ca^2+^ signals (Yu et al., 2005).

### Functions (b)—synthesis of Ins (1,3,4,5)*P*_4_

The possibility that Ins(1,3,4,5)*P*_4_ is a second messenger in its own right has been extensively discussed elsewhere (e.g. Irvine and Schell, 2001), and needs no expansion here. The only directly relevant recent information concerns our own attempts to gain evidence for the hypothesis (Irvine, 1989; Soriano and Banting, 1997; Irvine and Schell, 2001) that Ins(1,3,4,5)*P*_4_ acts to modify the structure of the e.r., thus regulating the mobilization of Ca^2+^ from the e.r. by Ins(1,4,5)*P*_3_. We have made concerted effort to seek evidence for such an effect on e.r. structure or movement using FRAP in several cell preparations, but have had to conclude either that the hypothesis is wrong, or that it cannot be detected by the technology we used (Brough et al., 2005a, b). There remain intriguing and strong data showing that Ins(1,3,4,5)*P*_4_ can exert profound influences on Ins(1,4,5)*P*_3_-regulated Ca^2+^ homeostasis (e.g. (Changya et al., 1989; Loomis-Husselbee et al., 1996), but the mechanism or universality of such responses remain unclear.

A very enticing indirect piece of evidence emerged recently from two independent reports that in the absence of the activity of IP_3_3KB, mice cannot mature their T cells, leading to immunocompromized animals (Pouillon et al., 2003; Wen et al., 2004). T lymphocyte Ca^2+^ signalling appeared superficially normal in both studies, and Wen et al. (2004) presented evidence that MAP kinase signalling might be altered. Both groups raised the issue that GAP1^IP4BP^, a putative Ins(1,3,4,5)*P*_4_ receptor (Cullen et al., 1995, 1997), which is very highly expressed in circulating lymphocytes (Lockyer et al., 1999), might be the protein involved, thus directly implicating Ins(1,3,4,5)*P*_4_ as the important factor missing in these mice.

Subject to some *caveats*, these findings are arguably the most compelling evidence yet that Ins(1,3,4,5)*P*_4_ is a physiological second messenger. The two *caveats* to this interpretation are, firstly that, as has been shown for many other proteins (including, e.g., the yeast Ins*P*_3_ multikinase (Dubois et al., 2000) and PI-PLC*γ* (Patterson et al., 2002)), IP_3_3KB may play a structural role, independent of its catlytic activity, as an indispensible member of a multi-protein complex. The second *caveat* is that the important missing component may be Ins*P*_6_ (or another higher inositol phosphate), rather than Ins(1,3,4,5)*P*_4_.

### Functions (c)—the first step in the synthesis of higher inositol phosphates

**Fig. 3:**
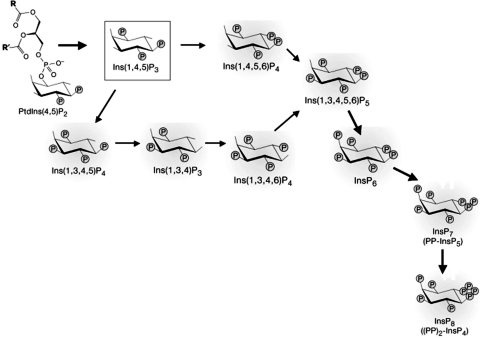
Pathways of Ins*P*_6_ synthesis. The figure depicts the two likely pathways for Ins*P*_6_ synthesis in mammlian cells. One route, the upper pathway (Odom et al., 2000), is found in yeast, may exist in mammals and does not involve any Ins(1,4,5)*P*_3_ 3-kinase activity. The lower route (Balla et al., 1989; Shears, 1989) has IP_3_3K as an essential first step.

The suggested synthesis pathway in mammalian cells for the higher inositol phosphates (functionally the most interesting of these are currently: Ins(3,4,5,6)*P*_4_, Ins(1,3,4,5,6)*P*_5_, Ins*P*_6_, Ins*P*_7_ and Ins*P*_8_ (see Irvine and Schell (2001); Shears (2001); Irvine (2005) for reviews)) was first pieced together largely by Shears and Balla and their co-workers in the 1980s (Balla et al., 1989; Shears, 1989). The cloning of inositol phosphate kinases in the following decades culminated in the identification of all the enzymes responsible for this pathway (Fig. 3), which goes as: Ins(1,4,5)*P*_3_ to Ins(1,3,4,5)*P*_4_ to Ins(1,3,4)*P*_3_ to Ins(1,3,4,6)*P*_4_ to Ins(1,3,4,5,6)*P*_5_ to Ins*P*_6_ to Ins*P*_7_ (Ins*P*_7_ kinase has yet to be cloned).

If this is indeed the major pathway by which animals make Ins*P*_6_, then acting as the first step on it is another potentially very important function for IP_3_3Ks. Elsewhere, the arguments for and against whether other pathways of Ins*P*_6_ synthesis are equally or more important are discussed (Irvine, 2005), and these would not be repeated here. In this context, the crucial issue is whether the ‘missing factor’ in IP_3_3KB knock-out mice (Pouillon et al., 2003; Wen et al., 2004) is Ins(1,3,4,5)*P*_4_ itself, or another component of the above pathway. As mice lacking ‘Ins*P*_3_ multikinase’ (Frederick et al., 2005) or Ins*P*_5_ 2-kinase (Verbsky et al., 2005) die at birth (compare the less severe phenotypes of IP_3_3K knock-out mice, above), superficially, it seems likely that IP_3_3K is not essential to higher inositol phosphate synthesis. We have recently set up a highly specific and sensitive mass assay for Ins*P*_6_ (RF I, AJL and MJS, unpublished), and by assessing the levels of Ins*P*_6_ in the above knock-out mice with this assay we hope to shed light on this issue.

## Summary

It is 20 yr since the Ins(1,4,5)*P*_3_ 3-kinase reaction was discovered, and this review summarizes and discusses some of the advances we have made in our understanding of the physiological significance of this reaction. The three major potential functions of Ins(1,4,5)*P*_3_ 3-kinases are discussed in the context of their localization and regulation, the possible functions of their product, Ins(1,3,4,5)*P*_4_ and their potential contribution to the synthesis of higher inositol polyphosphates.
